# Virtual field trips in hydrological field laboratories: The potential of virtual reality for conveying hydrological engineering content

**DOI:** 10.1007/s10639-022-11434-5

**Published:** 2022-11-28

**Authors:** Paula Farina Grosser, Zhongxin Xia, Jannik Alt, Uwe Rüppel, Britta Schmalz

**Affiliations:** 1 Department of Civil and Environmental Engineering, Chair of Engineering Hydrology and Water Management (ihwb), Technical University of Darmstadt, 64287 Darmstadt, Germany; 2 Department of Civil and Environmental Engineering, Institute of Numerical Methods and Informatics in Civil Engineering (IIB), Technical University of Darmstadt, 64287 Darmstadt, Germany

**Keywords:** Virtual reality, Augmented reality, Field trips, Hydrology, Education

## Abstract

With the start of the COVID-19 pandemic and the resulting contact restrictions, conducting field trips to hydrological research basins became close to impossible. Hydrological field knowledge is an essential part of hydrological education and research. In order to impart this knowledge to students of hydrological engineering subjects in times or situations where on-site exploration is not possible, the VR4Hydro tool was developed. VR4Hydro is a virtual reality platform built from 360° panoramas that allows users to interactively explore the Gersprenz River basin in Germany. The following study seeks to investigate the applicability of performing virtual field trips in the context of hydrological education by evaluating user experience. Sixteen students of the subject engineering hydrology were asked to document their experiences with VR4Hydro using a qualitative approach by answering a series of multiple-choice questions as well as long-answer text questions. The analysis and discussion of the results showed that virtual excursions generally met with great interest among users. The majority rated the virtual tour as a valuable addition to traditional teaching methods. All students found the tool particularly appealing in cases where external circumstances did not allow for a real excursion. The findings of this study show that the application of virtual field trips (VFT) in hydrological engineering can be a valuable supplement to real field trips to improve the interest and learning outcome of students.

## Introduction

Hydrological field laboratories play an important role in hydrological research and education (Schmalz et al., 2015, 2019). When studying hydrological processes or conducting water resources investigations, watersheds form the basic spatial unit (Ehret et al., 2014). Several universities, such as the University of Gothenburg (Rütting, 2021), the ETH Zürich (Hirschi, 2021) or the University of Natural Resources and Life Sciences in Vienna (Fürst et al., 2021) have designated research catchments for educational and research purposes. In 2016, the Chair of Engineering Hydrology and Water Management (ihwb) at the Technical University of Darmstadt established the Gersprenz Catchment in Germany as field laboratory (Schmalz & Kruse, 2019). Since then, the basin has been used in engineering hydrology education in addition to being the subject of many master- and bachelor theses. Further, the catchment is area of interest for various doctoral and research studies (David & Schmalz, 2020, 2021; Grosser & Schmalz, 2021; Kissel & Schmalz, 2020; Scholand & Schmalz, 2021). To provide a sound basis for research, the chair is collecting high-resolution measurement data and time series to complement existing long-term regional data. A field trip to the basin is offered each semester to give students an overall impression of the catchment, its topography, landscape and hydrologic characteristics, while familiarizing them with measurement techniques and equipment. With the onset of the COVID-19 pandemic in December 2019 and subsequent associated restrictions, such as contact limitations, the educational system was challenged massively (Daniel, 2020). These restrictions led to a cancellation of the hydrological field excursion, which is an essential part of the education in hydrology for students in environmental engineering at TU Darmstadt. Out of the necessity to overcome this deficit and to introduce students to the field laboratory in different ways, the VR4Hydro Project was born. VR4Hydro is a virtual reality platform based on open-source frameworks that allows users to interactively explore the Gersprenz river basin and to visualize hydrological data from field measurements. Apart from the need to find a substitute for the hydrological field trip due to COVID 19 restrictions, the use of virtual field trips (VFT) in teaching has gained importance in recent years due to the possibilities it offers. In general, the use of Virtual Reality (VR) and Augmented Reality (AR) is finding application across disciplines, particularly in engineering and science education e.g., Halabi (2020), Liou and Chang (2018), Stojšić et al. (2017), Hsu (2020). Numerous studies show that VR is enriching as a supplement to traditional teaching (Bos et al., 2021; Halabi, 2020; Hsu, 2020; Lin et al., 2017; Pantelidis, 2009; Stojšić et al., 2017). Some studies investigate the use of VR technology as a replacement or supplement for field trips in education. For instance, Cliffe (2017) conducted a theoretical study on the benefits and drawbacks to Virtual Field Trips (VFTs) in geoscience higher education. Stainfield et al. (2000) assessed the general effectiveness of digital interactive supplementary material such as reading material, websites or maps as supplements to real excursions. Bos et al. (2021) investigated the application of VR technologies for geography education and field trips to virtually introduce students into the landscape of a study area. In addition, the study focused on practicing and developing methodological techniques and identifying potential risks that might occur during a field trip. Evelpidou et al. (2021) developed a VFT for students in higher education with focus on geomorphology which is created using a combination of ArcGIS and Google Earth. Arrowsmith et al. (2005) developed a VFT of Grampians National Park in Australia for a mapping course in geospatial studies, mainly focussing on the impact of tourism on the environment. The 3D virtual environment was created at three scales, including a combination of satellite images, topographic maps and site-specific photographs.

In the field of hydrology, some applications of VR and AR exist. In 2014, Demir developed an interactive, web-based platform for the simulation of hydrological concepts and processes for teaching in civil- and environmental engineering as well as geography using a variety of different visualization techniques (Demir, 2014). The tool offers the possibility of realistically simulating flood scenarios and controlling them through various management options. Mirauda et al. (2017) introduced a mobile AR platform as a supportive tool for water management, which works in combination with the real environment and computer-generated data. It enables the presentation of non-visible details and objects to improve water monitoring activities and physical processes. Wolf et al. (2021) developed a 360° model used for a VFT to waterworks for students in environmental engineering and urban studies.

Only a few studies deal with VFT in hydrological engineering. Habib et al. (2010) developed a hydrological observation system in virtual reality, allowing for simultaneous acquisition of measurement data and creation of a process-based rainfall-runoff model in a catchment area. The tool was developed for its use in teaching hydrology as well as for the virtual re-enactment of an excursion. Besides, Kingston et al. (2012) assessed the implications of the use of VFTs in physical geography for hydrological education, using a mobile application for a Geographic Information System (GIS) based processing of spatial data in combination with field measurements.

Preliminary work in the field of hydrology dealing with VFTs focused primarily on the application of 3D models. The goal of the VR4Hydro project was therefore to combine 3D panoramas of the research catchment area with measurement and GIS data displayed in VR. Additional aerial footage was created with drone flights. The combination of different media types and data in VR allows students to gain impressions of the watershed's characteristics, such as basin size, topography, land use, water level, river discharge, etc. The platform can be used as a web application or in VR mode. User interaction takes place via keyboard, mouse, and touch inputs. For mobile devices, motion sensors support navigation through the panoramas. Switching between scenes is accomplished through hotspots. The cooperation project of the Chair of Engineering Hydrology and Water Management and the Institute of Numerical Methods and Informatics in Civil Engineering of the TU Darmstadt was financially supported by decentralized funds for quality assurance in teaching of the Department of Civil and Environmental Engineering. This paper aims at presenting the development and the functions of the VR4Hydro tool. Since virtual excursions specifically designed for teaching and research purposes in the field of hydrological engineering are still rarely represented in the scientific literature, this study intends to fill this gap.

Furthermore, most studies lack the evaluation of user experiences with the use of VFTs in hydrology. In this study, the user experience of students who used the tool for a virtual hydrological field trip is assessed by means of a survey. Moreover, the effectiveness of the introduction of VFT in water management and engineering hydrology education is evaluated on the basis of the research results. This paper summarizes the potential of using VFTs in hydrological engineering to improve quality and combine teaching and research.

## The field laboratory

**Fig. 1:**
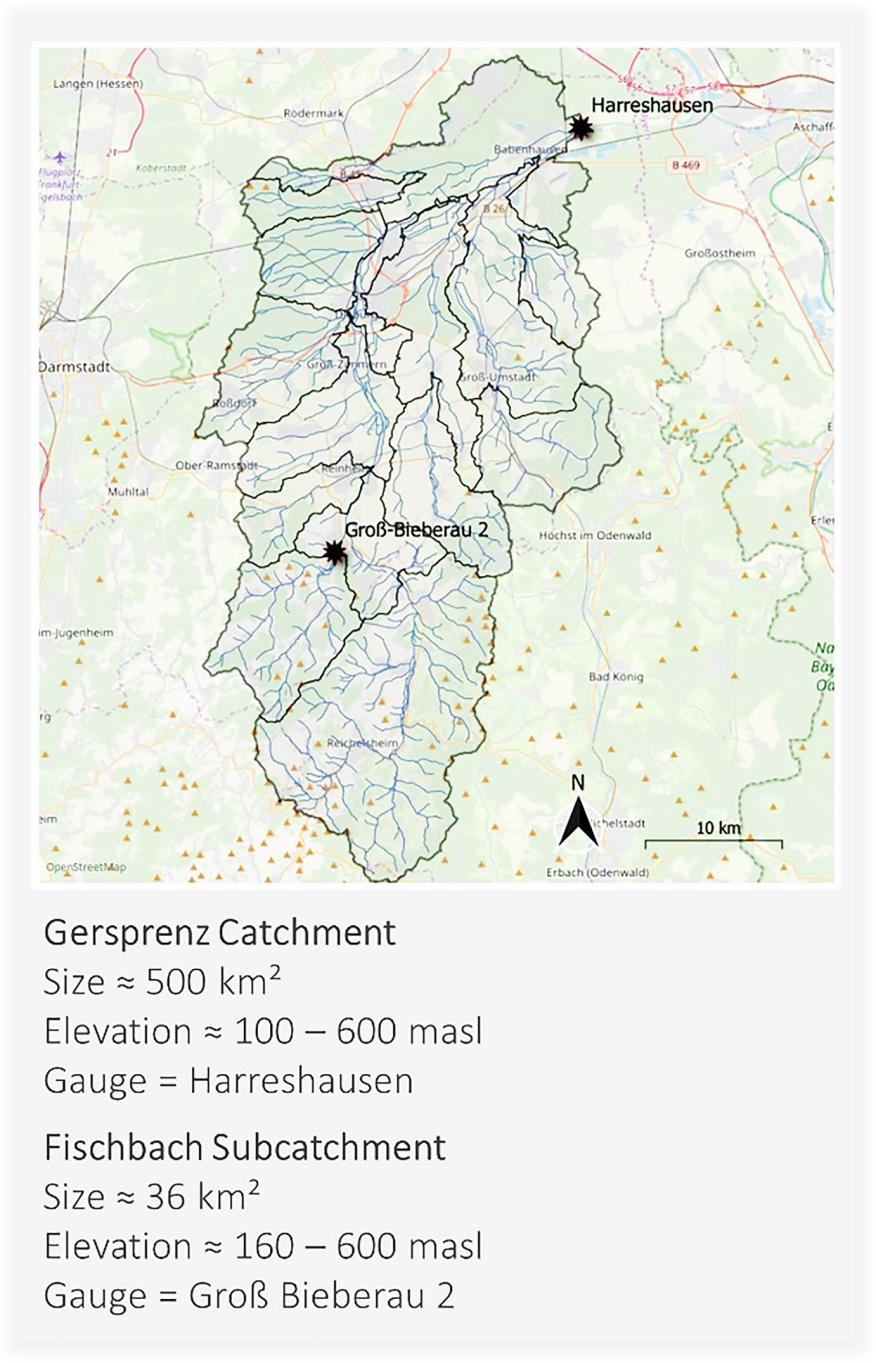
The Field Laboratory

With the new appointment of the ihwb chair in 2016, the Gersprenz catchment in southern Hesse, Germany, was selected as hydrological field laboratory. The total catchment has a size of approximately 500 km^2^. With the goal to conduct research on various spatial scales, the smaller sub-catchment of the Fischbach (approximately 36 km^2^) is further subject to measurement campaigns and data collection (Fig. 1).

The Gersprenz river flows into the Main River and thus is part of the river basin district Rhine. The southern part of the catchment, where also the Fischbach sub-catchment is located, covers the low mountain range area *Odenwald* with crystalline bedrock. In the central and northern parts, the catchment is hence defined by the hill country *Reinheimer Hügelland* and the *Untermainebene* (lower Main river plain). Unconsolidated rock soils made of sand, gravel and clay dominate here. The catchment encompasses mainly agricultural land use types, which add up to 48.3%. Forests rank second with 36.1%, while Settlements make up 12.6% of the land use coverage (Schmalz & Kruse, 2019). The mean discharge of the Gersprenz river for the time period 1980 to 2018 measures 3.08 m^3^/s, while the mean discharge for the same time period is 0.34 m^3^/s for the Fischbach (Grosser & Schmalz, 2021; HLNUG, 2019). Various larger and smaller tributaries flow into the Gersprenz. Further hydrological elements are ponds, mill channels and city/castle ditches. In addition, retention areas have been created for flood relief.

High-resolution measurement data and time series are continuously compiled for the field laboratory in addition to the long-term state data already available. Continuously measuring sensors are installed at 5 locations in the research catchment. At 10 stations measurement data is collected in a weekly measurement campaign. This intensive data collection and research activity, especially in the sub-catchment, which serves as small hydrological study areas, is used for:

- the understanding of hydrological processes,
- model application and development, and model testing for sensitivities and uncertainties,
- mapping the impact of climate and land use change, land management and water governance.

With the aim of creating a basis for more extensive learning processes and studies, VR4Hydro was established. The virtual tour is designed to familiarize users with the field laboratory and to demonstrate hydrological processes, landscape structures, topography and other catchment characteristics in addition to measurement techniques, equipment and data.

## VR4Hydro

###  Set-up

**Fig. 2:**
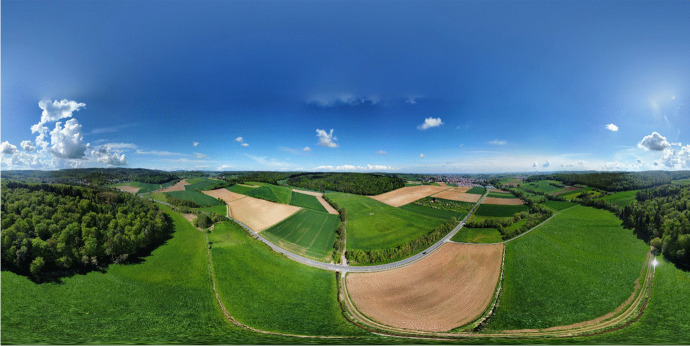
360° panorama of the O- and N- monitoring sites recorded by drone aerial photography

                                                                                                                                                                                                                                           Measuring  500 km^2^, the research catchment of the Gersprenz river is rather large. Creating a 3D model for the entire catchment area would have been overly time-consuming. For this reason, it was decided to present and display the river basin with the help of representative areas, namely the ihwb monitoring sites and the spring of the Fischbach stream. The objective was to illustrate these areas with 360° panoramas in order to create an overall portrayal of the site. The 360° panoramas cover the entire horizontal field of view and a vertical field of view of 180° (Fig. 2). Panoramic 360° VR can be explored within a browser or with the help of VR glasses by head movements. In agreement with Eiris Pereira et al. (2017), panoramic VR creates highly detailed, real, natural surroundings in which the user can immerse themselves, unlike common computer-generated simulations of an environment.

The VR4Hydro model includes 16 accessible stations which can be explored by the user. These hotspots are used to provide the user with the opportunity to obtain additional information about a particular scene. In the case of VR4Hydro the hotspots can be used to explore hydrological monitoring stations. In total, 16 hotspots are located in the catchment of the Gersprenz river, 14 of them in the sub-catchment of the Fischbach. Each site encompasses at least two panoramic photos: one is the aerial panorama of the region and the other is a panorama overlooking the riverbank. The aerial panorama contains a detailed record of features on the ground, such as topography, land use and the watercourse. In this project, the drone DJI Mini 2 was used to record the aerial panorama and videos (DJI, 2021). The Insta360 ONE X2 camera was used to create panoramas on the ground (INSTA360, 2021). Once the locations were decided upon, each panorama took only a few minutes to shoot. Sunrise and sunset proved to be the best times of day for taking these panoramic photos, as the objects in the landscape gained plasticity through lateral light.

**Fig. 3:**
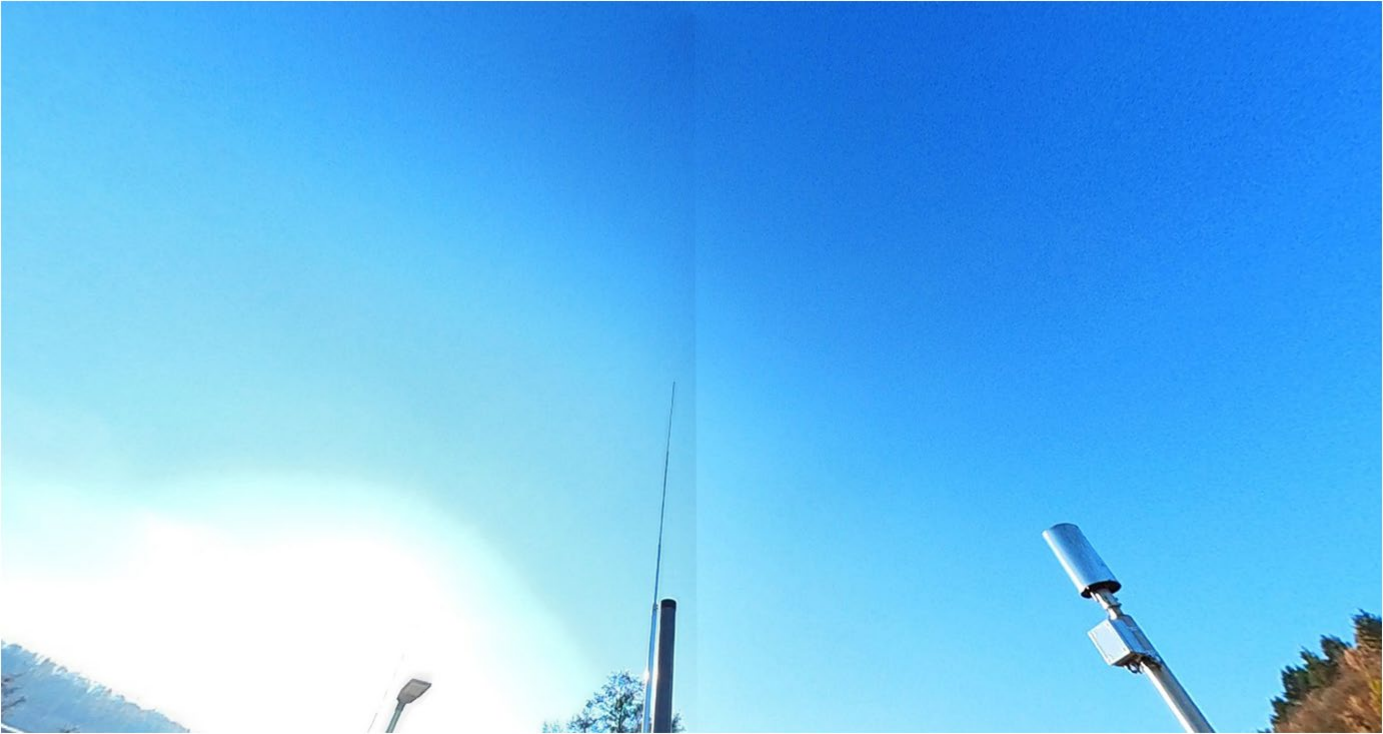
Parallax in the middle of 360° panorama

**Fig. 4:**
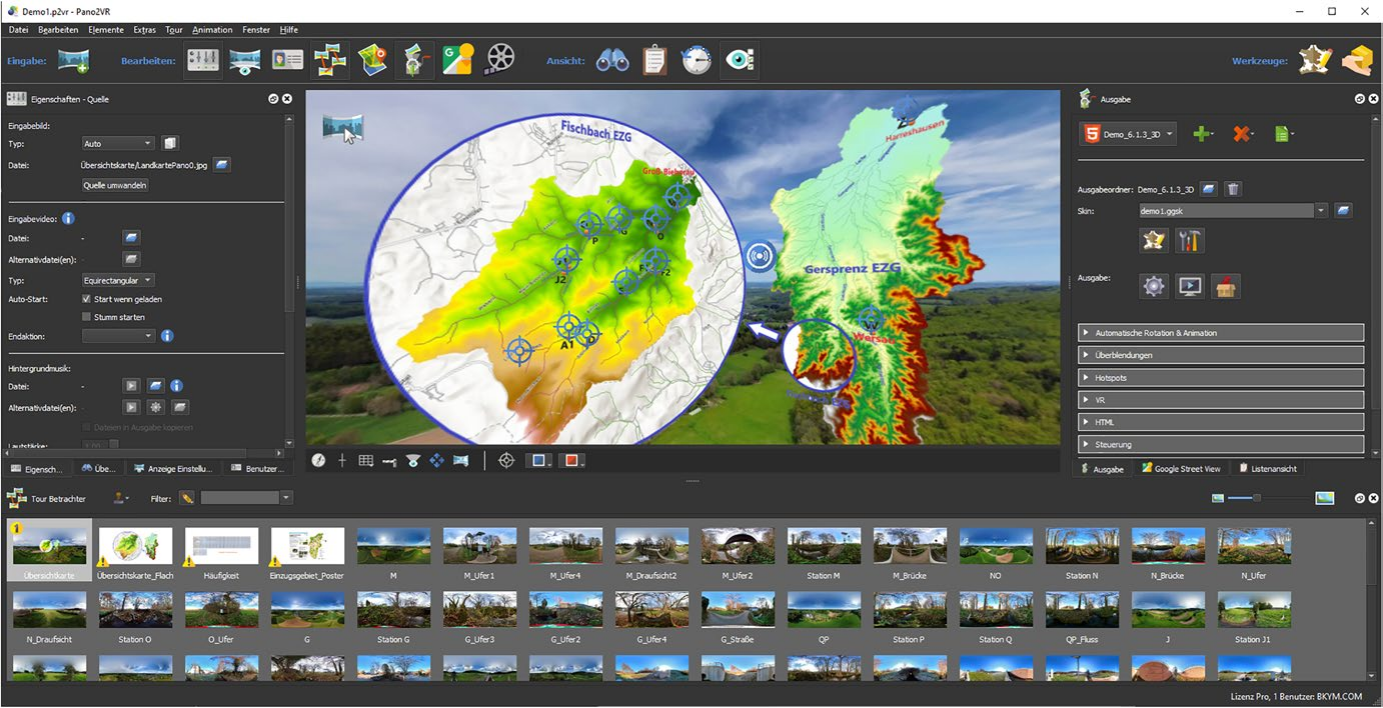
User interface of Pano2VR pro

The panoramas taken with these sorts of cameras are so-called stitched panoramas. They are created by connecting several photos with slightly overlapping fields of view to form a panoramic image (e.g., Zhang & Feng, 2014). Stitched panoramas contain a so-called parallax, an error which is caused by the difference of the angular position of two stationary points. An example is given in Fig. 3. The post-processing to remove these errors was done in Adobe Photoshop (Adobe, 2021). To assemble the panoramic images into an interactive virtual tour and to present this in a web browser, the program Pano2VR pro was used. Pano2VR pro is a virtual tour software that converts panoramic or 360° photos and videos into interactive experiences (PanoSociety, 2021). Figure 4 shows the user interface of Pano2VR pro.

**Fig. 5:**
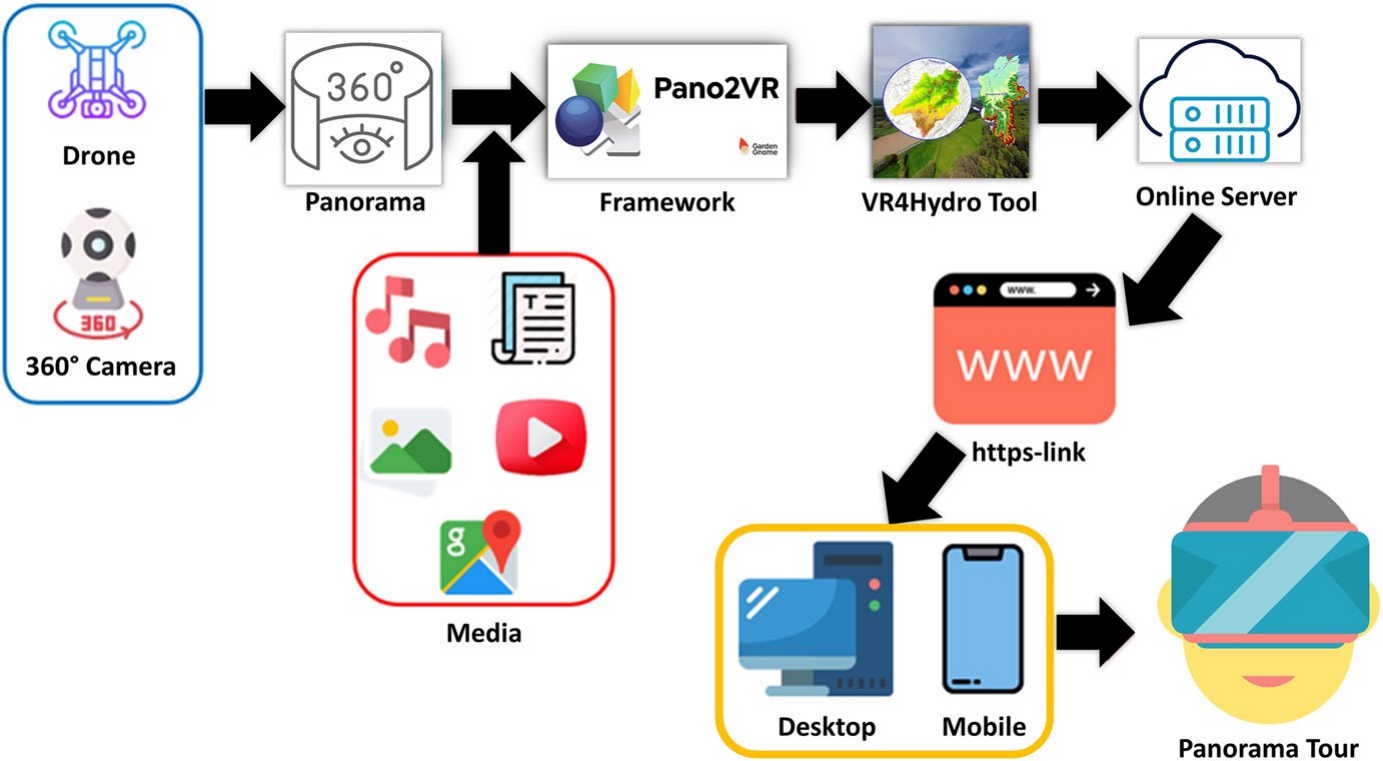
Summary of the Set-up process of the VR4Hydro tool

Within the program, hotspots with different functions were created to connect and open panoramas, pop up an information box, image or video or to open websites. Various media such as background music, drone videos, geographical locations, etc. were embedded in the panorama to enhance the model content. The actions of the hotspots were adapted to the needs of the tool and the icons and images were edited in the skin editor. A skin is a graphical element that is layered over the panorama and can include images, text, video, sounds, buttons and toolbars. After compiling and processing, the VR4Hydro model was stored on an online server. The html file of the VR4Hydro model is the main entry point. The server can generate an HTTPS URL corresponding to this html file. This HTTPS URL is an internet communication protocol that protects the integrity and confidentiality of data between the user's browser and the site (SoftEd Systems, 2021). This means through the HTTPS-Link the model can be opened on a desktop computer or smartphone. The whole set-up is summarized in Fig. 5.

### Tool functionalities

VR4Hydro is an interactive platform for students and researchers in the field of environmental engineering and hydrology that aims to virtually familiarise the user with the research basin of the Gersprenz River. The tool was developed for the digital enrichment of prospective lessons, the creation of digital teaching materials and the delivery of terrain knowledge in times of the COVID-19 pandemic. It is a user-friendly tool to complement field trips to the Gersprenz catchment area. VR4Hydro is intended as a cost-effective and simple solution. By using inexpensive cardboards, impressions on area characteristics such as catchment size, topography, land use, watercourses, discharge etc. can be easily conveyed.

Since 2016, the chair of the ihwb is collecting high-resolution measurement data and time series at 15 measurement sites in the Gersprenz catchment. 360° videos were recorded at each of these stations, including the source of the Fischbach, forming the 3D virtual environment. This provides a realistic impression of the catchment morphology, land use and land cover as well as the geometry of the watercourse.

VR4Hydro can operate via a web browser on personal computers and mobile devices, as well as in VR mode on smartphones. The tool was also equipped with additional information on the measurement methods, land use and geography of the catchment, as well as other aspects in the form of commented videos, drone videos, images, text boxes, charts and audio files. These elements are implemented in the virtual landscape and provide additional information about the stations and the corresponding devices.

The 360° videos, drone recordings, as well as a compass, aim to improve the user’s spatial orientation. In addition, overview maps are placed throughout the virtual space to give users quick access if they do get lost. High-resolution hydrological data, such as time series of discharge and water temperature can be retrieved at each station. The user can move between the locations by gaze, hand controller, or clicking a hotspot. Hotspots are overlays on the 360° media. They are located within the landscape and linked to the corresponding stations or additional information.

**Fig. 6:**
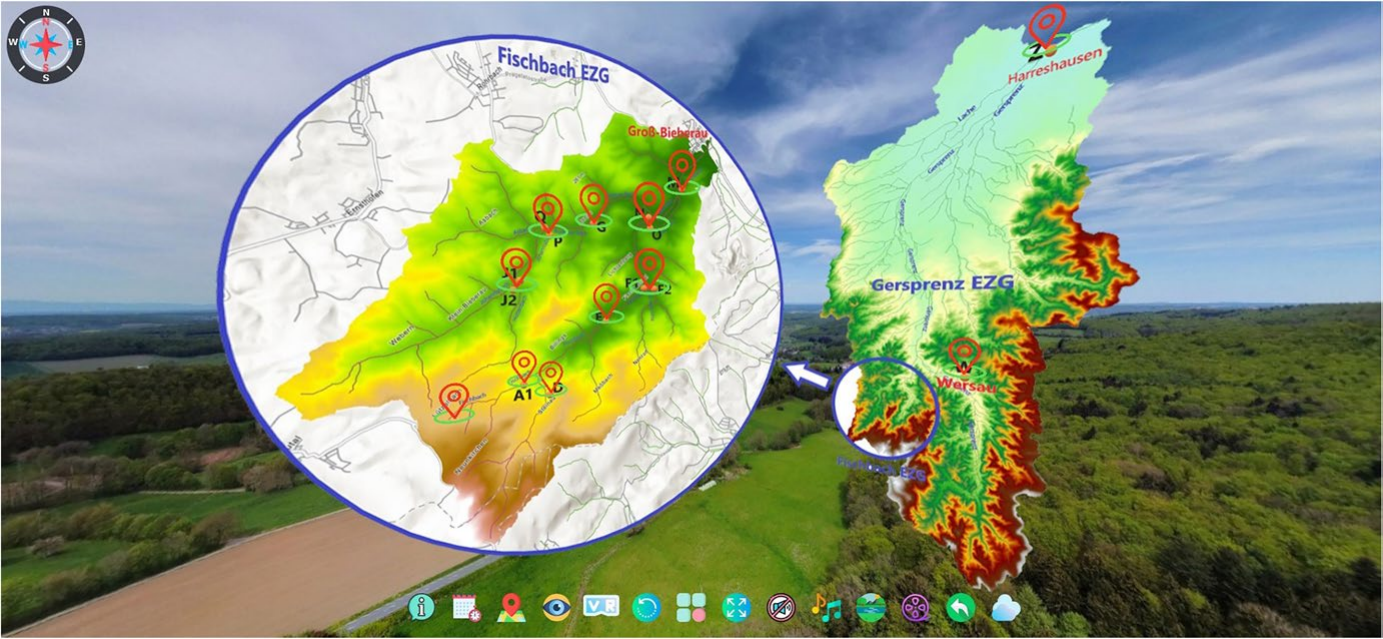
Start screen of the VR4Hydro platform including an overview map of the Gersprenz and Fischbach catchments. Note that EZG is the abbreviation for the German term Einzugsgebiet (catchment area)

**Fig. 7:**
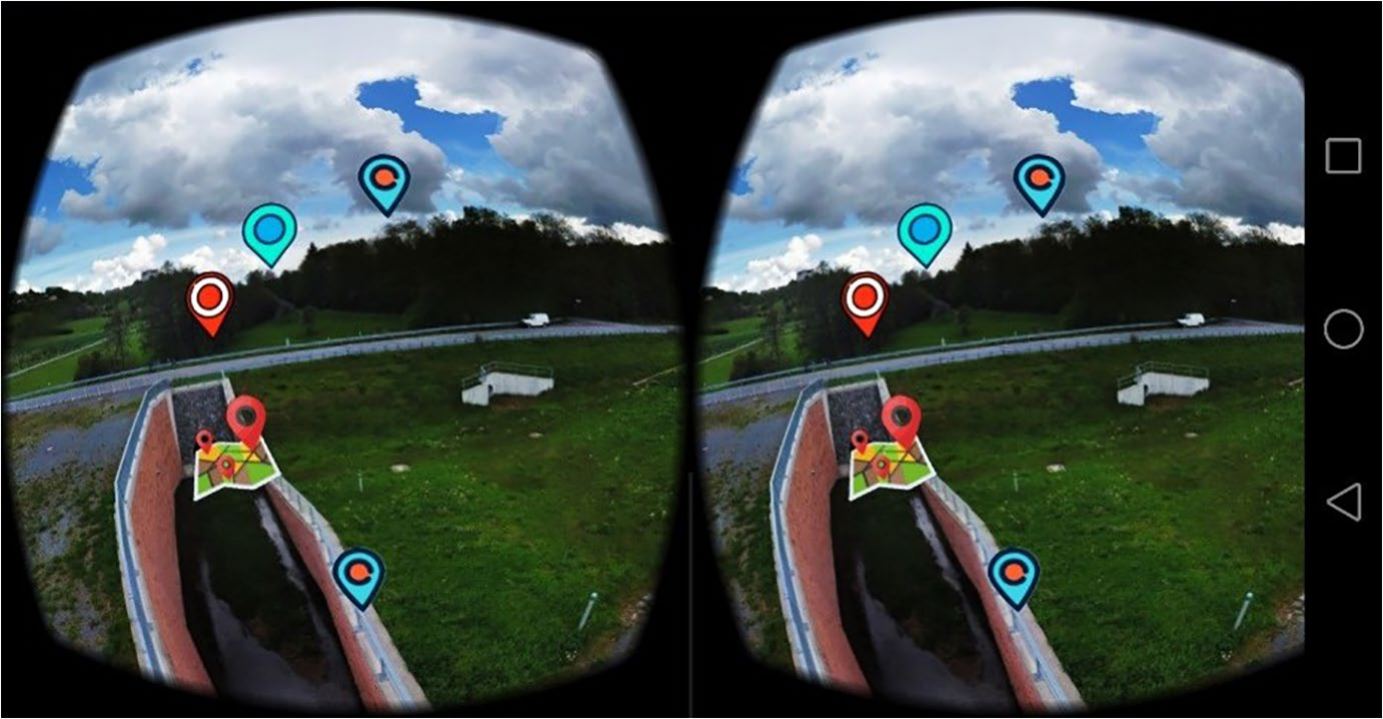
Virtual Reality Mode

Figure 6 shows the homepage of the platform, featuring the ubication of the stations within the catchment area. The catchment area of the Gersprenz is shown on the right, the sub-catchment area of the Fischbach on the left. The locations are illustrated by landmark symbols, which are linked to the corresponding stations. The user can navigate through the virtual catchment using mouse, keyboard or touchscreen – in VR mode the navigation is supported by sensors of the mobile device (Fig. 7). The menu bar on the bottom of the screen as shown in Fig. 6 provides different audio, video and navigation options that enable access to information concerning the research catchment. Table 1 presents the different menu icons in detail and shortly explains their functions.

**Table 1:**
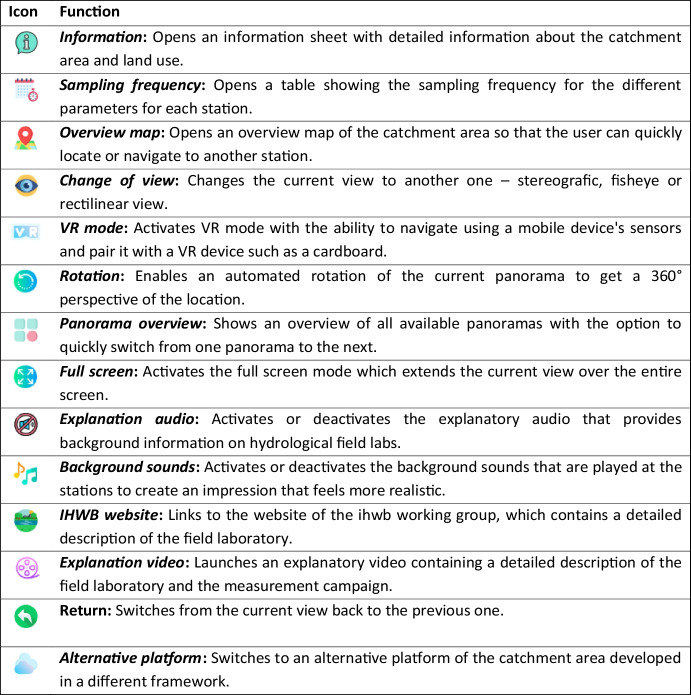
Description of the different menu icons and their functions

**Fig. 8:**
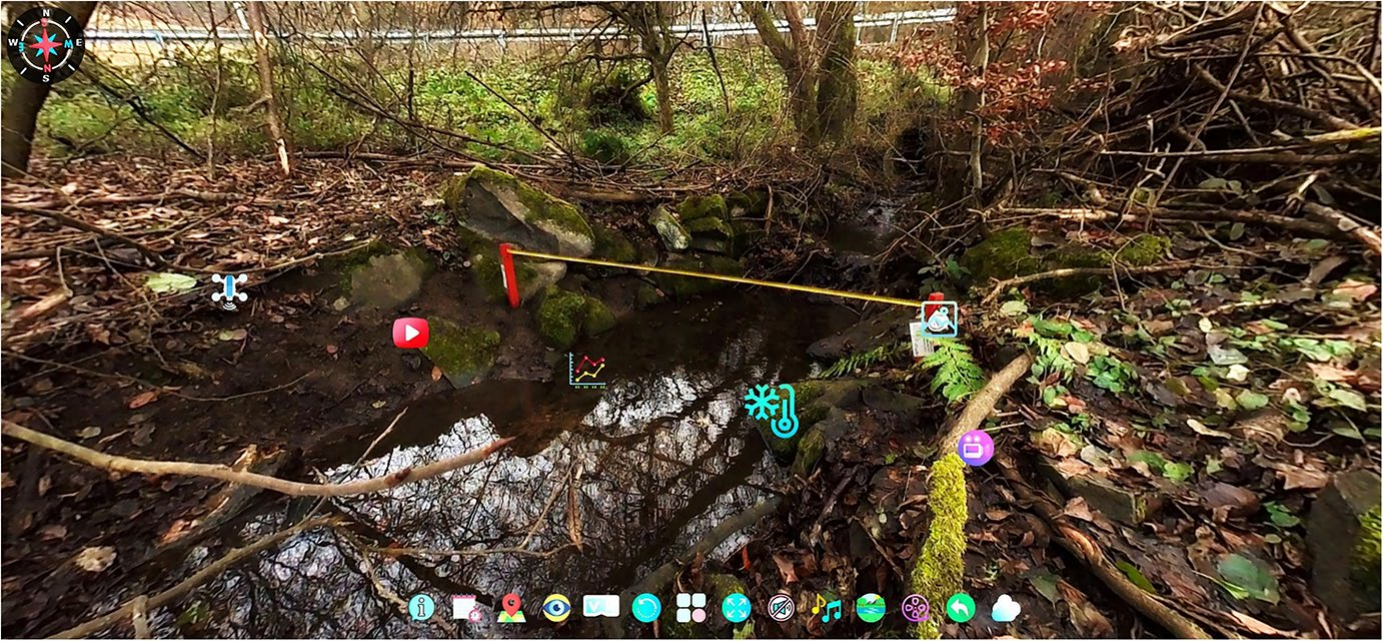
View of the station A1 with several AR elements for further information and data

**Fig. 9:**
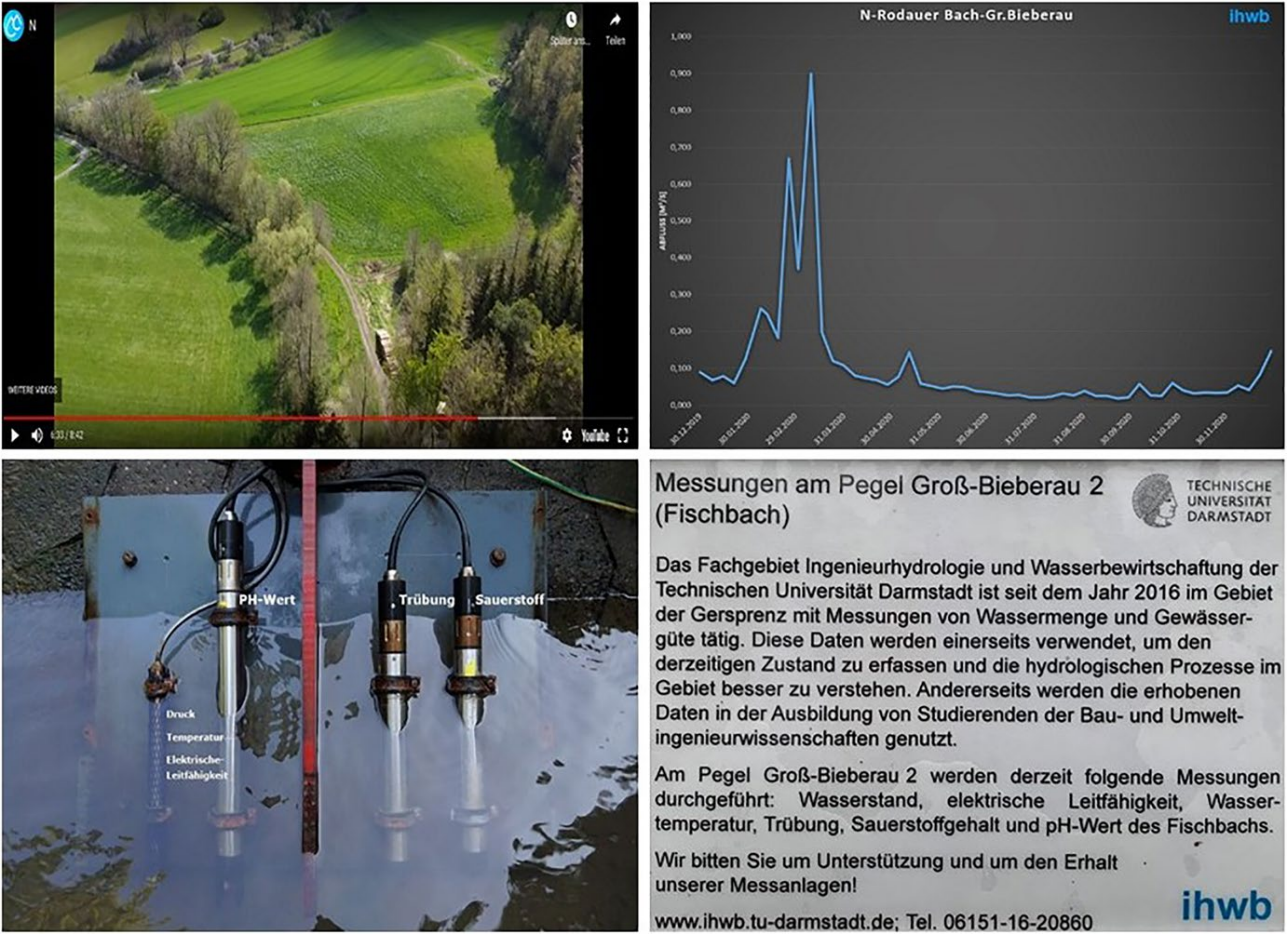
Examples for additional information provided by AR elements: drone video (top left), stream discharge (top right), water quality sensors (bottom left), information table (bottom right). Note that the tool was initially created for educational purposes and local stakeholders in a German-speaking environment. Efforts will be made to make the tool available in English soon

The user can access the measuring stations by activating the respective hotspot. Initially, a bird's eye view opens. Clicking on the blue location arrow takes the user to the measurement station. An example of a location in the Fischbach is given in Fig. 8. The symbols and functions of the different elements, which can be found at the stations, are presented in Table 2 on the next page. Examples on additional information are given in Fig. 9.

**Table 2:**
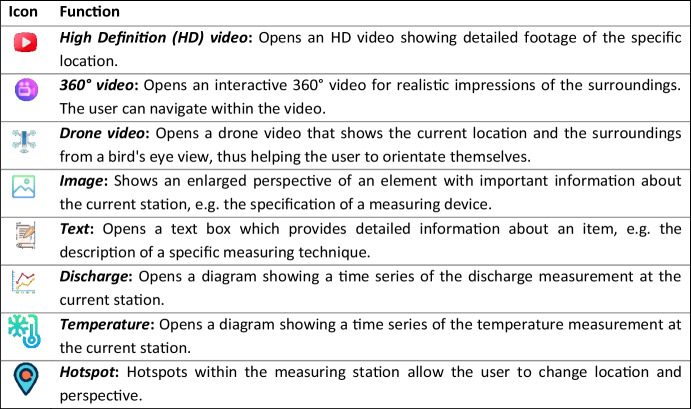
Description of the different AR elements.

## Evaluation 

To evaluate the tool, a user survey was conducted with students of civil and environmental engineering at the TU Darmstadt. The procedure is briefly outlined below, followed by a presentation of the results. Subsequently, the results will be further discussed.

### Method

The user survey is based on 16 questions and was developed and conducted using Google Forms (Google, 2021). The first 12 questions of the questionnaire were multiple-choice questions, which were evaluated based on the percentage of the chosen answer options in relation to the total number of answers for each question. The last four permit answers in the form of sentences ("long-answer text"). Three of the qualitative survey questions were evaluated by categorizing the answers into five groups: Audio/sound, visualization, operation, functionality, and information content. The other question was evaluated by categorizing the answers into two groups describing reasons for and against virtual excursions. Half of the questions are related to technical or operational matters concerning VR4Hydro. The other half deals with learning outcomes or suggestions for improvement.

The survey was conducted with 16 students in the subject Engineering Hydrology II. This Master module covers hydrological topics such as rainfall-runoff processes, erosion and soil loss, ecohydrology, impacts of land use and climate change on water resources, and water management strategies etc. Throughout the module, the students work on a project in the Gersprenz catchment, more specifically at the Wersau gauging station. The students are to complete various tasks for the subcatchment upstream of the gauge. The assignments include calculating the potential evapotranspiration in this area, as well as determining the effective precipitation and the resulting direct runoff for the community of Wersau and the catchment as a whole.

Within this module the tool served to gain a first impression of the project area. Within the scope of one lesson (1.5 h), the VR4Hydro project was briefly introduced by means of a Power Point presentation. Only the most important features of the tool and its operation were explained in a time frame of about 15 min. The students were then left to explore the VR4Hydro platform independantly. The questionnaire for the survey was sent out at the same time as the link to the tool. However, the students were asked to start answering the questions at the earliest after 20 min had passed. The introduction, trial phase and survey took place on the video conference platform Zoom. Throughout the whole time, two experts were available to respond to questions and assist with technical issues if necessary. After completion of the survey, the answers were downloaded and evaluated. All students answered the questions comprehensively. With this evaluation method, each user was requested to perform an individual and independent assessment of his or her experience with the functionality and learning success of the VR4Hydro tool.

### Results

**Fig. 10:**
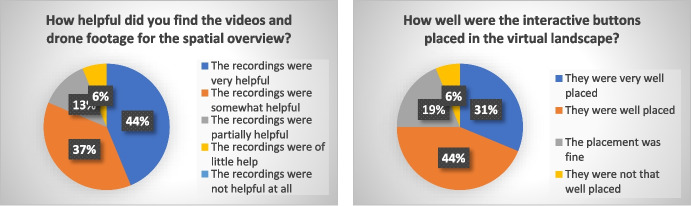
Results of the survey conducted with 16 students of the subject Engineering Hydrology. The questions above are related to technical or operational matters concerning the VR4Hydro tool

**Fig. 11:**
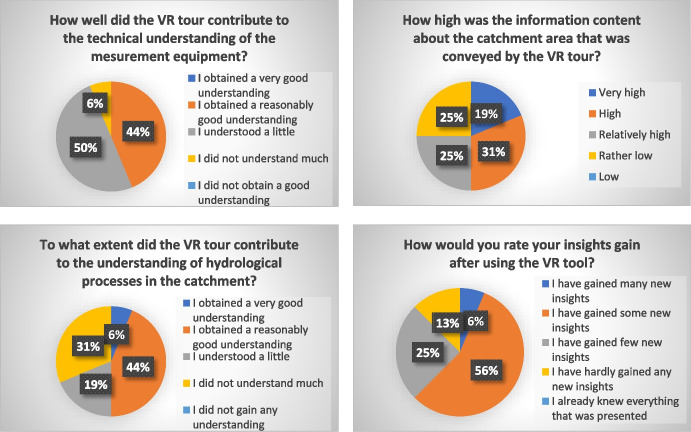
Results of the survey conducted with 16 students of the subject Engineering Hydrology. The questions above refer to learning outcomes and content to be conveyed by the VR4Hydro tool

In this subchapter, the results of the survey are presented. All answers to multiple choice questions are displayed as charts in percentages. Figure 10 shows the results of the questions concerning technical or operational aspects of the VR4Hydro tool. The first question relates to how the tool was accessed—whether students used the platform in a browser or in VR mode. The results show a clear preference for using the platform via a browser: 81% of students used browser access, while the remaining 19% used both the browser and VR mode on a mobile device. None of the students used the tool in VR mode only. However, it is important to note here that VR glasses were not required for participation and thus only those students who had such a device available were able to experience the tool in VR mode. Looking at the feedback on the operational experience with the tool, 50% of the users liked the menu and another 37% even found it very appealing. 13% of the users found it rather appealing. Further, 88% of the survey participants found the navigation in the virtual catchment easy or very easy and did not have any difficulties to navigate to a specific location or find a particular piece of information. The remaining 12% had some difficulties with the navigation and retrieving specific information within the tool. Overall, the feedback concerning the operation of the tool was predominantly positive. Positive feedback was also received for the use of videos and drone footage to support spatial overview; for 81% of users, the footage was helpful or very helpful. For 13% of the participants the footage was at least partially helpful. Only one person found the footage of little use. The placement of the interactive buttons in the virtual landscape, e.g., the hotspots used to navigate to a specific location, were very well- or well-placed according to 75% of the participants. 19% of the students found the placement of the buttons fine; only one student did not completely agree with the placement. Overall, responses from students who participated in the survey indicate a mostly positive experience using the VR4Hydro tool from both a technical and operational perspective. The graphs in Fig. 11 display the results of the multiple-choice questions related to information content or learning outcomes. 44% of the participants obtained a reasonably good understanding of the measurement processes and equipment. Half of the participants stated that they understood a little. One person did not understand much of the technical aspects of the measuring. More than half of the participants found the information content transmitted by the tool about the catchment area itself high or relatively high. The rest of the students stated that they found the conveyed catchment information rather low or even low. Finally, the tool contributed to the understanding of hydrological processes in the catchment to 69% of the students. The other 31% did not understand much. It can be seen that the answers concerning the learning content and outcome had a higher spread than the answers dealing with technical issues. Nevertheless, more than half of the participants gained many or some new insights and a quarter of the students gained a few new insights using the tool. Only two out of 16 people stated that they hardly gained any new insights using VR4Hydro.

**Fig. 12:**
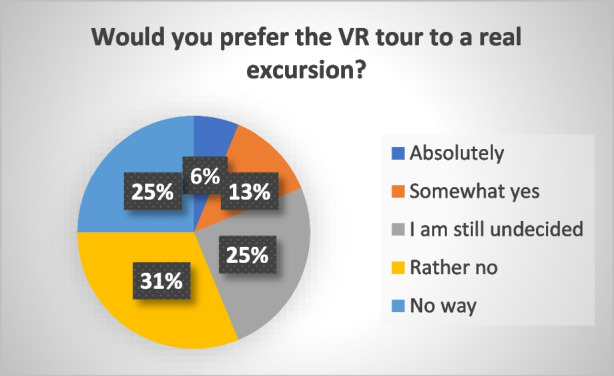
Results of the survey conducted with 16 students of the subject Engineering Hydrology. The above question evaluates whether or not students prefer a virtual field trip to a real field trip

Finally, the students were asked whether they would prefer the virtual excursion over a real one. The answers are presented in Fig. 12. Furthermore, the students were asked to explain their answers qualitatively. Only one survey respondent answered that they would absolutely prefer the VR tour to a real excursion, on the grounds that a virtual tour saves more time. Two people answered with somewhat yes, stating that with a virtual tour it is easier to quickly visit several places of the catchment, while during a real excursion the time needed to travel from one gauge to another makes it impossible to visit all monitoring sites. A quarter of the students are still undecided. 56% of the students lean to (rather) not replace real excursions with virtual ones. They however, explain in their answer sentences that they think that the virtual tour is a great supplement to the real one. About 70% of the students state that they would welcome a virtual excursion when for certain reasons a real excursion is not possible. For instance, in case of distance or a pandemic. In summary, the reasons why students would prefer a virtual tour to a real tour are that it provides a better spatial overview and orientation, it is convenient and time-efficient because it avoids the need to travel to the catchment area, and it provides a time-independent access to the VR tour and thus the catchment and measuring stations. Reasons that were mentioned against the VR tour included limited access to information (during an excursion it is possible to ask questions to a teacher or professor, while in a virtual tour one can only access the presented information), limited impressions on the environment and the spatial context of the measurement stations. In addition, three students think that it is easier to remember information that was conveyed during a real excursion than during a virtual excursion. Table 3 provides an overview of the survey participants' responses on the reasons for or against virtual field trips compared to real field trips.

**Table 3:** Reasons that the students mentioned for and against using VR4Hydro

Reasons for VR excursion	Reasons against VR excursion
• Time saving and Simple	• Only selective insights into the catchment area are possible
• Can be accessed from anywhere and thus no journey is necessary	• Limited view of surroundings and surrounding villages
• Covid-19 pandemic	• Lack of spatial coherence/ understanding distances is difficult
• Conveys a good overview of the catchment	• Information is not memorized as well
• Spatial context more visible	• Rotation within the VR produces nausea
• Free movement and choice of location in the research basin	• Limited availability of data and information
• Observation and comparison of several measuring points	• It not possible to ask questions
• Access to the tool also after the excursion and at any time	• Limited consideration of individual measuring devices

Further questions were asked to determine the student’s user experience with the tool. The question of what the students liked best about the VR tour was answered by 12 out of 16 students. The answers are summarized in Table 4. The answers were categorized into five topics: sound, visualization, operation, functionality and information content. Several students stated that they enjoyed the sounds and background music incorporated in the tool as well as the detailed graphical representation. Besides, the participants found the navigation within the tool intuitive, as it is easy to operate. In addition, the fact that the area is easily accessible and that the tool gives a good spatial impression of the catchment  was positively commented upon. Furthermore, the students were asked what kind of problems they encountered during their use of VR4Hydro. This question was addressed by 12 out of 16 students. The answers were categorized in Sound, Visualization and Functionality and are summarized in Table 5. One student mentioned that the explanatory audio was drowned by background noises. However, there is the possibility to switch off either background music or explanatory audio, in order to avoid overlapping. In addition, some users had problems with localizing buttons or hotspots. One student complained about experiencing nausea during the VR tour. Two students had problems exiting the VR mode when using Firefox. It was found that this problem was eliminated when other browsers were used (e.g., Google Chrome or Microsoft’s Internet Explorer).

**Table 4:** Evaluation of the most responsive aspects of the VR tour

*What did you like most about the virtual tour?*				
*Sounds*	*Visualization*	*Operation*	*Functionality*	*Information content*
• Background music and audios	• 360° views	• Easy navigation	• Easy operation	• Quick overview of the catchment
• Music with option to turn off	• Love for Detail	• Easy movement back and forth between gauging stations	• New types of working methods	• Outlet Hydrographs
	• Graphical representation			• Good spatial impression of the catchment
	• High quality Pictures and videos			
	• Underwater video			
	• Bird's eye view			
	• Drone footage

**Table 5:** Evaluation of problems encountered during the virtual tour

*Did you encounter any problems during the virtual tour that affected its use?*		
*Audio*	*Visualization*	*Functionality*
• Explanation audio is drowned out by background noise	• Difficulty finding some buttons	• Continuous rotation after deactivation (2x)
	• Nausea when used without VR glasses	• Calling up the frequencies of measurements only possible from the starting page
	• 360 videos too short	• Exiting the VR model was not possible when using Firefox

Finally, the students were consulted on improvement suggestions for the tour. 10 out of 16 students answered this question thoroughly. The suggestions were categorized into visualization, functionality and information content and summarized in Table 6. Some interesting suggestions included showing and visualizing the flow direction of the water for a better orientation, implementing longer 360° video, as for some the time to explore within the video was insufficient, to extend the options of movement in the catchment area and enable tracking the user’s location in “GPS-style” on a miniature map. Furthermore, some users suggested increasing the amount of provided information. Several students would have liked to be able to access more information on specific measuring devices and techniques.

**Table 6:** Evaluation of improvement suggestions

*Do you have any suggestions for improving the virtual tour?*		
*Visualization*	*Functionality*	*Information content*
• Fixed placement of the exit button	• Diagrams of discharge and temperature should be easier to close	• Hydrographs not continuous
• Show flow direction of the waters	• Extend movement options at respective stations and enable tracking of the on a mini-map	• Provide more measurement data at the Wersau gauge
• Uniform quality of the videos		• Increase information content
• Smoother video quality		• Provide additional measured values
• Longer 360° videos		• Reference to the source of the measurement data
• Do not place the camera in the area of the riparian vegetation		• Display and describe more measuring instruments
• Clearer distinction of the buttons of explanatory audio and background music		• Background information on specific locations
		• More information about measuring instruments with extra menu function
		• More detailed explanation of the various measuring devices and procedures through additional videos

## Discussion

The results of the VR4Hydro user survey helped to clearly depict the students' perceptions of virtual excursions and their advantages and disadvantages. Furthermore, it was possible to deduce the the effectiveness of the introduction of VFTs in water management and education in the field of engineering hydrology from the evaluation results. Importantly, for the majority of respondents, the fast, barrier-free accessibility of the virtual catchment tour outweighed the disadvantages as a major advantage. The quick accessibility makes it possible to use the virtual tour flexibly in teaching as well as for homework and theses. These findings are in line with previous studies by Demir (2014), Cliffe (2017), Stainfield et al. (2000), Pugsley et al., 2022, Evelpidou et al. (2021) and Wolf et al. (2021), who found that VR platforms, as a supplement to field trips or field courses, provide access independent of time and space. This generally allows students to work autonomously and interactively.

The results of the survey further showed that the virtual excursion was found to be particularly interesting when traveling to the research area was difficult or impossible due to certain reasons, such as large spatial distances, disabilities or contact restrictions, as in the context of a pandemic. These findings are supported by studies by Cliffe (2017), Stainfield et al. (2000), Pugsley et al. (2022) and Evelpidou et al. (2021), that pointed out several advantages of virtual field trips over real field trips. E.g., a VFT allows inclusion of students with physical limitations, especially when difficult terrain conditions occur. Besides, the virtual platform is free of charge and users can participate without having to cover costs for traveling etc. This allows all students to participate, despite their financial situation. In general, it can be said that participation in a virtual excursion is less dependent on external factors than participation of a real excursion. Among other things, for example, from unfavourable weather conditions or seasons.

The time and cost saving aspect was found to be an advantage. Not only from the user side but also from the developer’s side. As there is no need for additional staff, personnel and material, the educational institution can also save costs (Chang & Liou, 2018). Also, Pugsley et al. (2022) pointed out the financial inclusivity of VFTs compared to a physical field trip due to the omission of travel expenses. The case study of Wolf et al. (2021) underlines the financial aspect by using a cost-efficient software environment for the development of the VFT.

Furthermore, it was considered advantageous by many students that not only one measuring site can be visited exemplarily, but that all measuring sites in the catchment area can be accessed as required, the individual choice of locations playing an important role for many. In addition, VR4Hydro makes it easy to get a spatial overview of the Gersprenz catchment and allows comparisons to be made between different measuring stations. Navigation with the help of the map was found to facilitate orientation within the study area. In this way, the VFT allows the user to get to know the area from different perspectives. In the following, examples of statements made by the interviewed students are quoted. Note that the statements were translated from German into English, whereby it was intended to keep the statement as close as possible to the original.

*“(…) Another advantage is that you can see a spatial context better with the tool, because you can look at the area from above and quickly jump back and forth between the gauges and so the information that you get at the individual gauges can be more easily spatially classified. (…) It is easier and I can choose many places myself. (…)”*

*“A real excursion still gives better impressions of ONE station, but not such a good overview of the whole area. (…)”*

In line with previous studies, the results show that the use of VR offers an interesting alternative to traditional teaching methods due to the many possibilities it provides. Several students pointed out that they enjoyed the supplementary material in the tool, such as drone aerial videos, background music, audio descriptions etc. Several options for additional digital interactive media are also described by Stainfield et al. (2000).

Halabi (2020) found that the use of VR in teaching improved means of communication, problem solving skills, learning outcomes and finally also students’ grades. Also, studies by Chang and Liou (2018) and Stojšić et al. (2017) found that learning success highly increased when using VR in education, among others due to the variation and the interactive features it offers. This also includes the possibility of visualizing measurement data. The high level of motivation conveyed by student participation and student involvement in feedback in this study, indicates increased student engagement and interest in the experience of VFTs. Complementing findings by Bos et al. (2021), Pantelidis (2009) and Hsu (2020) that found that the addition of VR to traditional teaching increased the motivation and learning readiness of students. Further enriching and supplementing regular education, while fostering student’s creativity and curiosity (Lin et al., 2017; Stainfield et al., 2000). Also, Wolf et al. (2021) reported positive results from students’ experience with VFT in terms of motivation, emotion and usability. Finally, an advantage of a VFT is that it can be adapted, extended or enhanced to the required needs at any time, given that expert knowledge is available (Cliffe, 2017).

In addition to commendable aspects, some points of criticism were identified by the survey. Some of the questioned students perceived the absence of an accompanying expert or supervisor as a major disadvantage, as it may not be possible to ask questions or discuss topics of interest during a VFT:

*“(…), additional explanations, which are given spontaneously during a real excursion, are of course omitted. (…) during an excursion you can ask questions about individual things, look at things closer and from all sides. (…)”*

*“(…) Especially to the measuring instruments or boxes and gauge houses, which were shown here in the tool ""only"" as a photo, you would ask questions during an excursion, how it looks inside and what is exactly in the box etc....”*

These findings are confirmed by (Cliffe, 2017), who states that no or limited direct communication is possible during a VFT. This makes it difficult for a team spirit to develop between the group of students. Similar findings were reported by Pugsley et al. (2022) who pointed out the loss of social cohesion. Other survey participants stated that they found it inconvenient or strange that the haptic and olfactory senses are not addressed during a VFT, concluding that a real excursion may be more memorable, as one notices more about the surroundings. This aspect is consistent with the study of Pugsley et al. (2022), who found the "loss of travel and outdoor experiences and the loss of traditional field education" as a disadvantage of VFTs through a survey of students. Insights of the catchment are limited during a virtual excursion and for some it was harder to grasp the spatial context:

*“On a virtual tour you will only see one or a few point shots of the area. In a real excursion you see more of the surroundings and especially the surrounding villages, which are located in the catchment of the respective river / stream. (…) You get a rough overview in the Virtual Tour, but the distances between the gauges and the location itself are quite difficult to estimate.”*

One student complained about dizziness while using the tool in VR mode. Bos et al. (2021), found that the usage of a VR headset might cause motion sickness, addressing a similar point in their study. Even though, personnel, material and travel costs are saved during the excursion the latter study also pointed out that the creation of the tool requires financial resources, not to mention the necessary technical knowledge to apply this technology to a specific teaching purpose. Also, studies by Cliffe (2017) and Kingston et al. (2012) point out that building a VR platform or tool requires a profound technical expertise and financial resources. In addition, the user of the tool must have access to a stable internet connection and certain minimum hardware requirements.

Summing up, for most users the advantages of virtual excursions outweighed the disadvantages; some suggested that they generally prefer real excursions. However, all users agreed that for cases where a real excursion is not possible and as a basic supplement to regular classes, the virtual excursion is an excellent tool. This result falls in line with findings of Stainfield et al. (2000), Kingston et al. (2012), Pugsley et al. (2022), Evelpidou et al. (2021) and Wolf et al. (2021) that state that VFT can always be seen as an addition to real field trips rather than a replacement. Overall, positive feedback from students clearly prevailed. Therefore, it may be concluded that VFTs are an effective tool for enhancing teaching and learning while improving student's motivation and engagement. In general, the approach was positively evaluated and well received as a new way to deliver teaching content and exploring the research catchment.

## Conclusions, Limitations and Future Work

**Fig. 13:**
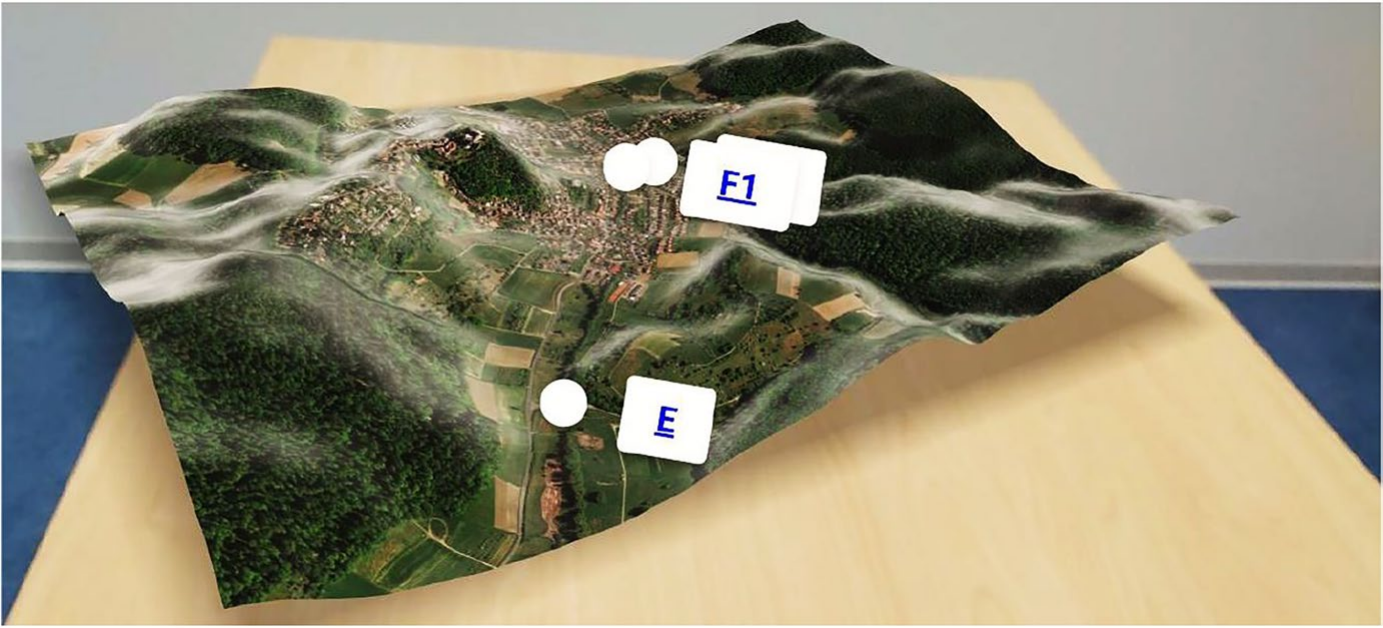
A Digital Terrain Model representing part of the research basin is shown as AR in a classroom

The survey conducted among hydrological engineering students as part of this study helped to evaluate the user experience with the VR4Hydro tool. Even though the positive feedback of the surveyed students on the VR4Hydro tool predominates, some critical points were raised that can be addressed or improved by enhancing or expanding the tool. One aspect that was mentioned by some of the questioned students was the difficulty of grasping or understanding spatial context while virtually exploring the Gersprenz research basin. To improve the spatial understanding of the users it would be possible to add digital terrain models to the tool. Furthermore, hydrological processes within the catchment area may be visualized by means of simulations. This was done in a study by Demir (2014) in which an interactive environment for teaching hydrological processes and impacts of human activities and interventions within a watershed was developed. Allowing, for example, the possibility of realistically simulating flood scenarios and controlling them through various management options. Besides, Habib et al. (2010) developed a hydrological observation system in virtual reality, visualizing hydrological processes as time series and spatial distributions. Both of the latter VR environments were designed as 3D models. Taking it a step further would be to bring the model directly into the classroom with the help of AR. This was already tested in the scope of the VR4Hydro project. An example of how showing a part of the catchment as Digital Terrain Model in a classroom could look like is given in Fig. 13.

The tool offers many possibilities in teaching and educational hydrology. However, the use of the tool can be extended and applied in various realms. Within the VR4Hydro project, we aim to utilize the tool also in the context of scientific research and the communication and visualization of research results. As of now, VR4Hydro can already be applied to introduce visiting scientists or interested parties to the research basin. Summing up there are many possibilities within this novel field of research and communication. The study has shown that conducting a VFT in an environment which is not solely computer simulated, but based on 360° panoramas, allows the users to explore a detailed, natural, realistic surrounding. Using additional media, such as background sounds allows an even more wholesome experience and increases the exploratory spirit. An immediately available access for students and researchers to the virtual field trip is always an effective complement to a real field trip and can even sometimes replace it. This study showed that a VFT is a very good way of introducing a research basin, such as the Gersprenz field laboratory. It enables the user to get an overall impression of the catchment, its landscape and topography, as well as hydrological features, while familiarizing them with the measurement techniques and equipment.

With the development of the VR4Hydro tool, the study has shown that by providing an interactive platform for virtual excursions, an effective complement to physical excursions can be created in the educational and scientific field of hydrological engineering. The survey results showed that the interactivity of the tool created a high level of motivation among the respondents to explore and learn about the Research Basin. Therefore, it can be concluded that the provision of virtual excursions in education improves the overall quality of teaching and probably increases the learning success. However, it was also found that for several users a virtual excursion could never fully substitute a real excursion. Nevertheless, the VFT was shown to be a valid alternative and supplement in traditional teaching and especially welcomed when external factors did not favour a real excursion. The results of this study are an important contribution to the knowledge about the effectiveness and applicability of virtual excursions in the field of hydrological teaching and research.

## Data Availability

The datasets produced and analyzed in this study are available on request from the corresponding author.

## Abbreviations

*AR*: Augmented Reality
*EZG*: Einzugsgebiet (Catchment)
*GIS*: Geographic Information System
*GPS*: Global Positioning System
*HD*: High Definition
*ihwb*: Fachgebiet Ingenieurhydrologie und Wasserbewirtschaftung (Chair of Engineering Hydrology and Water Management)
*VFT*: Virtual Field Trip
*VR*: Virtual Reality
